# DeepARV: ensemble deep learning to predict drug-drug interaction of clinical relevance with antiretroviral therapy

**DOI:** 10.1038/s41540-024-00374-0

**Published:** 2024-05-06

**Authors:** Thao Pham, Mohamed Ghafoor, Sandra Grañana-Castillo, Catia Marzolini, Sara Gibbons, Saye Khoo, Justin Chiong, Dennis Wang, Marco Siccardi

**Affiliations:** 1 Institute of Systems, Molecular & Integrative Biology, Department of Pharmacology and Therapeutics, University of Liverpool, Liverpool, UK; 2 Department of Computer Science, University of Liverpool, Liverpool, UK; 3 Department of Infectious Diseases and Hospital Epidemiology, Departments of Medicine and Clinical Research, University Hospital Basel, University of Basel, Basel, Switzerland; 4 National Heart and Lung Institute, Imperial College London, London, UK; 5 Singapore Institute for Clinical Sciences, Agency for Science, Technology and Research (A*STAR), Singapore, Republic of Singapore

**Keywords:** Clinical pharmacology, Molecular biology, Cheminformatics

## Abstract

Drug-drug interaction (DDI) may result in clinical toxicity or treatment failure of antiretroviral therapy (ARV) or comedications. Despite the high number of possible drug combinations, only a limited number of clinical DDI studies are conducted. Computational prediction of DDIs could provide key evidence for the rational management of complex therapies. Our study aimed to assess the potential of deep learning approaches to predict DDIs of clinical relevance between ARVs and comedications. DDI severity grading between 30,142 drug pairs was extracted from the Liverpool HIV Drug Interaction database. Two feature construction techniques were employed: 1) drug similarity profiles by comparing Morgan fingerprints, and 2) embeddings from SMILES of each drug via ChemBERTa, a transformer-based model. We developed DeepARV-Sim and DeepARV-ChemBERTa to predict four categories of DDI: i) Red: drugs should not be co-administered, ii) Amber: interaction of potential clinical relevance manageable by monitoring/dose adjustment, iii) Yellow: interaction of weak relevance and iv) Green: no expected interaction. The imbalance in the distribution of DDI severity grades was addressed by undersampling and applying ensemble learning. DeepARV-Sim and DeepARV-ChemBERTa predicted clinically relevant DDI between ARVs and comedications with a weighted mean balanced accuracy of 0.729 ± 0.012 and 0.776 ± 0.011, respectively. DeepARV-Sim and DeepARV-ChemBERTa have the potential to leverage molecular structures associated with DDI risks and reduce DDI class imbalance, effectively increasing the predictive ability on clinically relevant DDIs. This approach could be developed for identifying high-risk pairing of drugs, enhancing the screening process, and targeting DDIs to study in clinical drug development.

## Introduction

Drug-drug interactions (DDIs) represent an important issue in the drug development process and complicate the clinical management of antiretroviral therapy (ARV)^1,2^. DDIs may occur when combining drugs where the effect of one drug alters the exposure (pharmacokinetic interaction) or the effect (pharmacodynamic interaction) of other drug(s), potentially resulting in adverse drug events or loss of efficacy. People living with HIV (PLWH) often present comorbidities requiring the concurrent use of multiple drugs thereby increasing the risk of DDIs outcome^3^.

Obtaining data through clinical DDI studies between all possible ARVs and drug combinations is highly challenging due to ethical, time and cost constraints^4^. Therefore, the evaluation of the risk associated with DDIs is mostly conducted using a mechanistic approach (i.e., elimination pathway and inhibitory/inducing effects of drugs) or based on expert opinion. More recently, physiological-based pharmacokinetic modelling (PBPK), an approach combining drug in vitro data and population physiology, has demonstrated an accurate predictive power to simulate clinically relevant yet unstudied DDI scenarios. PBPK guided dose recommendations have been approved in several drug labels as an alternative to real-world studies^5^. However, this method requires extensive mathematical modelling of relevant physicochemical and physiological processes and relies on the availability of experimental data, which is often time consuming and therefore cannot be readily applied to guide the management of DDIs in clinical practice.

Due to the importance of DDIs throughout drug development and for the clinical management of PLWH, efficient computational methods for predicting DDI risks are in need. Currently, the cutting-edge approach to this problem is via machine learning^6–9^, which is a branch of artificial intelligence that uses algorithms to extract patterns from given data to make predictions. Deep learning, a sub-field of machine learning, is inspired by the human neural network that offers powerful tools to generalise learning by mapping the artificial neurons between the given input and output data^10^. Transformer-based models are a type of neural network architecture that learns context and semantic information within sequential data via self-attention mechanism, gaining significant popularity for its effectiveness in capturing complex patterns/relationships within a sequence^11^. These models, such as BERT^12^, are often adopted in transfer learning scenarios, where a transformer model is pretrained on a large dataset using self-supervised learning tasks. The goal of the pretraining phase is to leverage the learned features and representations from that task which can subsequently be fine-tuned for specific downstream tasks. Designed for applications in cheminformatics, ChemBERTa^13^, pre-trained on 10 million compounds from PubChem, serves as a robust tool for molecular representations and holds potential for further refinement for DDI prediction tasks.

On the other hand, the concept of structural similarity, whereby molecules that are structurally similar are likely to have similar properties, has been employed and proven to be useful by substantially supporting evidence in the field of cheminformatics, such as QSAR/QSPR (quantitative structure activity relationship/quantitative structure property relationship) methods. Studies^14–20^ have expanded this concept to predict DDIs, assuming that if drug A has a DDI with drug B and drug A has a similar structure with drug C, it is likely that drug C establishes a similar DDI with drug B.

In the past decade, deep learning has gained growing interest where several algorithms have been developed to allow a more efficient integration of drug similarity features as input and predicted DDIs on a larger scale with high accuracy such as studies by Rohani and Eslahchi^21^ and Lin et al. ^22^ However, the DDI predictions were binary (presence or absence of DDI) which may not be sufficient to guide clinicians manage DDIs. On the other hand, Ryu et al. ^23^ has proposed a computational framework utilising molecular structural similarity to output 86 DDIs using causal mechanism types based on DrugBank’s descriptive database. Zitnik et al. ^24^ has developed an approach for modelling up to 964 side effects as a result of DDIs. Although these methods have provided advanced pharmacological knowledge in DDIs and various implications for adverse drug events, the predictions of DDI need to be refined to better predict the clinical relevance.

The University of Liverpool has developed a well-established resource to describe and classify DDIs between ARVs and a wide range of comedications (including prescribed and over-the-counter medicines, herbals and vitamin supplements, and recreational drugs), https://www.hiv-druginteractions.org. Clinical recommendations are based on a ‘traffic light’ system, with each recommendation accompanied by an assessment of the quality of evidence^25^ in a system which has some similarity with the principles of the Grading of Recommendations Assessment, Development and Evaluation System (GRADE). The database is broadly used in clinical practice to screen DDIs with ARVs. However, the curation process requires an extensive literature search, a large volume of detailed drug information and expert opinion which could be labour-intensive and time-consuming.

**Fig. 1:**
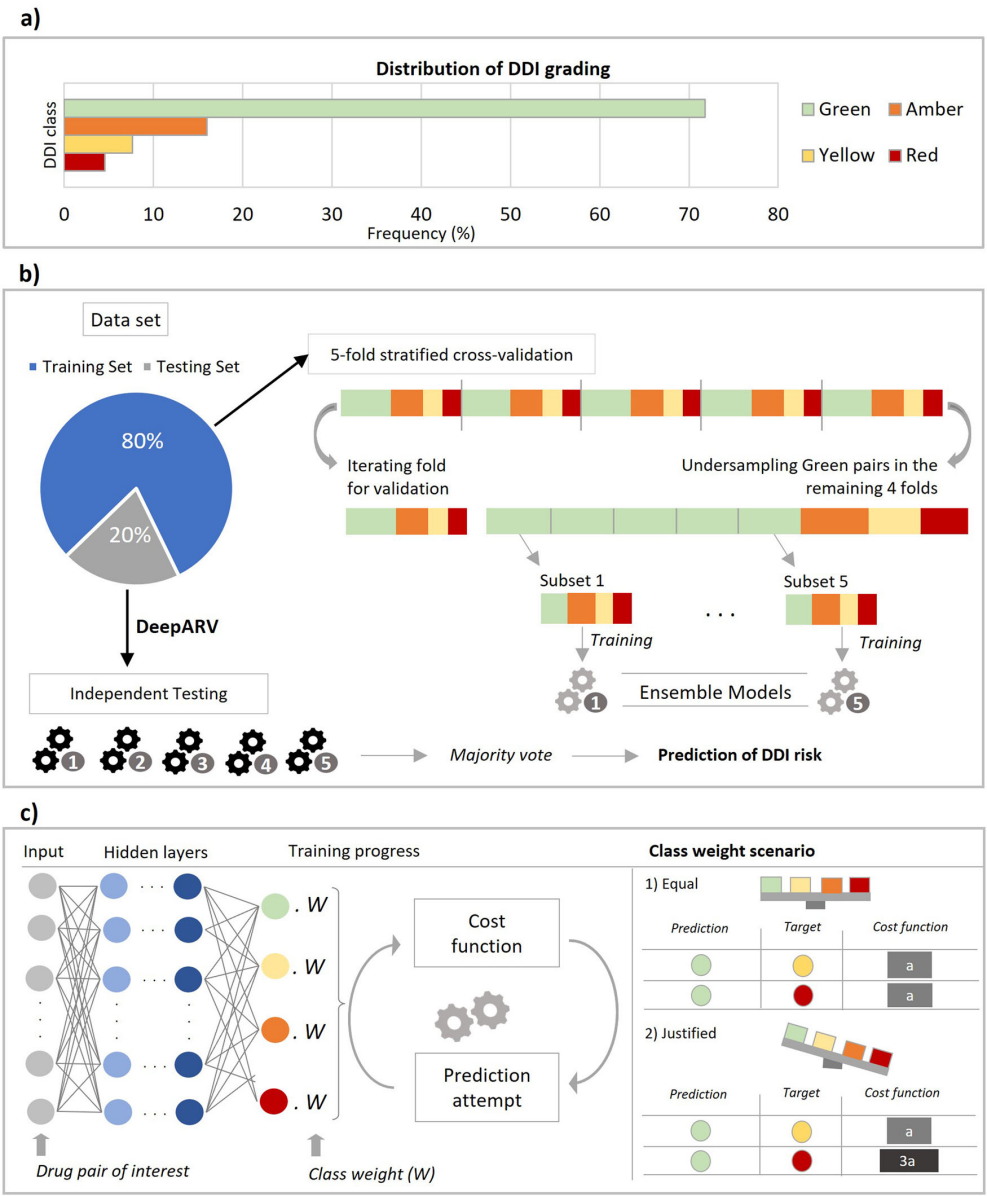
DeepARV framework to overcome imbalance challenge for improved prediction of four severity DDI gradings. **a** Skewed distribution of the DDI grading dataset. **b** Data sampling methods included undersampling combined with five ensemble models to split the dataset to five subsets by reducing the size of the majority class (Green DDI class), forming DeepARV. **c** Algorithmic method of adjusting class weight to increase the cost matrix of Red category and so its importance, thus penalising the misidentification against the Red DDI pairs the most and reducing the likelihood of misclassification of this instance in the future prediction.

Here, we propose two deep learning approaches called DeepARVs that build on the University of Liverpool HIV Drug Interaction database and aim to predict clinical DDI risk between ARVs and commonly used comedications. There are four DDI grading categories: 1) Green – No clinically significant interaction expected. 2) Yellow – Potential interaction of weak clinical relevance for which additional action/monitoring or dosage adjustment is not required. 3) Amber – Potential clinically relevant interaction that can be managed by clinical monitoring, alteration of drug dosage or timing of administration. 4) Red – These drugs should not be co-administered as they may cause a deleterious effect (e.g., loss of efficacy or toxicity of the ARV drug or co-administered drug). DeepARV framework included sampling and algorithmic adjusted weight techniques to overcome the imbalance challenge formed by the skewed distribution among DDI grading categories (Fig. 1). In addition, our study adopts two distinct input feature construction strategies for a given drug: 1) generating a similarity profile through molecular fingerprints, namely DeepARV-Sim, and 2) employing the advanced transformer-based model, ChemBERTa-2, as a molecular feature extractor, namely DeepARV-ChemBERTa.

## Result

### Molecular structural similarity analysis

**Fig. 2:**
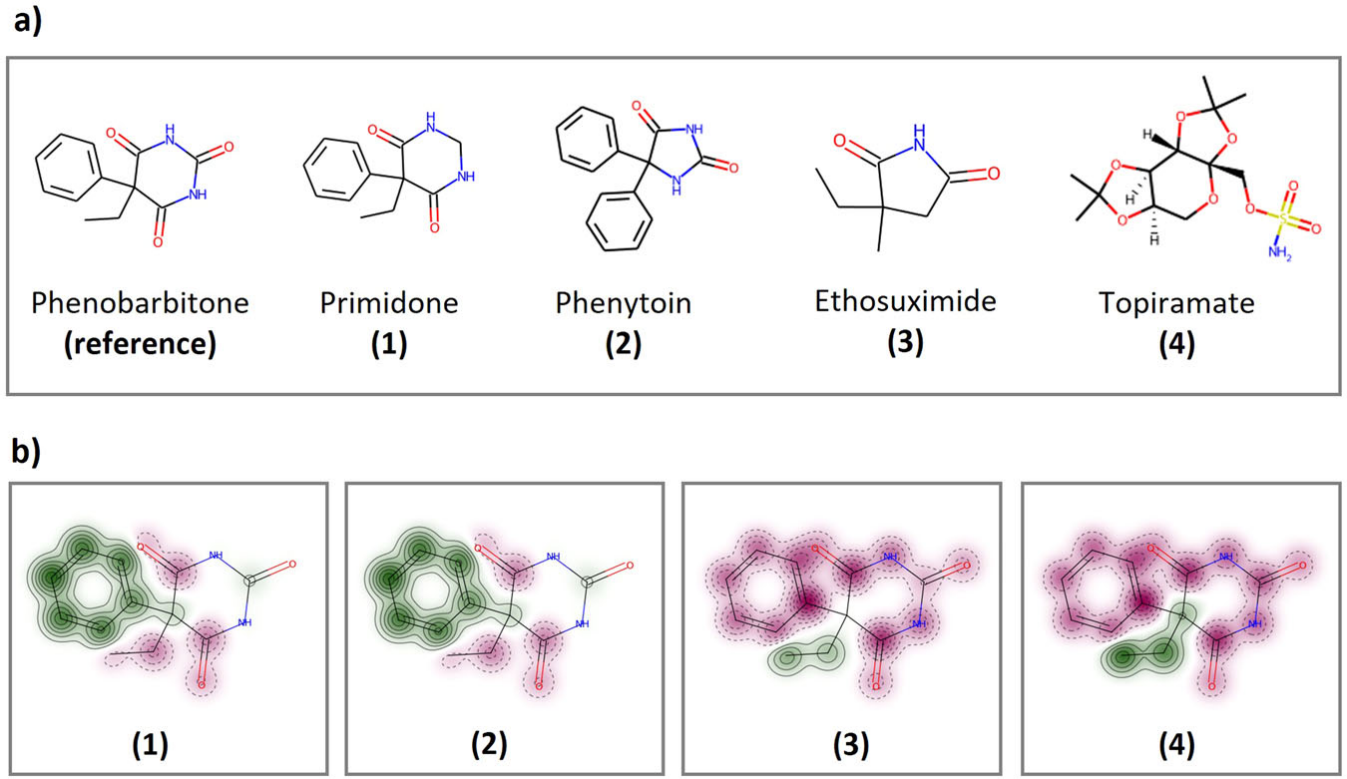
An illustrative example of molecular similarity maps for anticonvulsant comedication. **a** Reference compound is phenobarbitone. Test compounds include primidone (1), phenytoin (2), ethosuximide (3) and topiramate (4). **b** Similarity maps of a reference molecule compared to test compounds are generated via RDKit with the specification of Morgan fingerprint and Tanimoto similarity metric. Colour scheme indicates: removing green area bits decreases similarity (i.e. positive difference), removing pink area bits increases similarity (i.e. negative difference), no change in similarity in neutral area.

Structural similarity-based approach for DDI prediction could be derived from the *Similar Property Principle*^26^, which states that molecules that are structurally similar are likely to have similar properties. Measure of molecular similarity has two main components: 1) the descriptor to characterise the molecular structure of drug pairs of interest (also known as fingerprint), and 2) the similarity coefficient to quantify the degree of resemblance between two molecules. Our study employed Morgan fingerprint^27^ using *RDKit*, which is a hashed topological algorithm that assigns a numeric identifier to all the nearest neighbours within each atom (radius = 2, typically between 0 and 3 bonds)^28^. As a result, all identifier representations of each drug are hashed to a fixed-length binary fingerprint (*n* = 1024 bits). The Tanimoto coefficient was applied to compute a similarity score for each drug pair based on the fingerprint. Figure 2 provides molecular similarity maps of anticonvulsant comedication as an illustrative example (phenobarbitone – reference; primidone, phenytoin, ethosuximide and topiramate – test molecules). We found that DDI patterns between ARVs and comedications were more similar when a target and a query comedication were more structurally similar, example is provided in Supplementary Table 1.

### DeepARV accurately predicts DDI risks

The optimal architecture of DeepARV-Sim was composed of four hidden layers with {1024, 512, 256, 128} number of neurons respectively, ReLu activation function and the Adam optimiser. The optimal architecture of DeepARV-ChemBERTa was comprised of two hidden layers with {256, 128} number of neurons respectively, Tanh activation function and the Adam optimiser. During the training of both models, Green DDI category was under-sampled to five subsets and the remaining DDI categories were kept the same frequency in each subset. The class weight balance of {0.688, 1.448, 0.692, 2.429} for {Green, Yellow, Amber, Red} respectively was calculated using Eq. 1 where the final weight of each DDI category was inversely proportionate to the number of samples within that category. A stratified 5-fold cross-validation was performed on the dataset of 25,039 drug pairs (80% of the whole data) in which the dataset was divided into five equal-sized folds while preserving the same distribution of DDI risks in each fold. The process involved five iterations where 4-fold of data (*n* = 19,230 drug pairs) was applied for training and the remaining 1-fold for evaluation. DeepARV-Sim achieved a weighted average macro of 0.868 ± 0.011 accuracy, 0.811 ± 0.007 balanced accuracy, 0.757 ± 0.018 f1-score, 0.809 ± 0.006 precision, 0.737 ± 0.023 sensitivity (recall), and 0.885 ± 0.009 specificity across the evaluation folds (Supplementary Fig. 1). DeepARV-ChemBERTa yielded a higher weighted average macro of 0.904 ± 0.005 accuracy, 0.878 ± 0.003 balanced accuracy, 0.823 ± 0.009 f1-score, 0.868 ± 0.003 precision, 0.809 ± 0.01 sensitivity, and 0.948 ± 0.003 specificity (Supplementary Fig. 1).

**Fig. 3:**
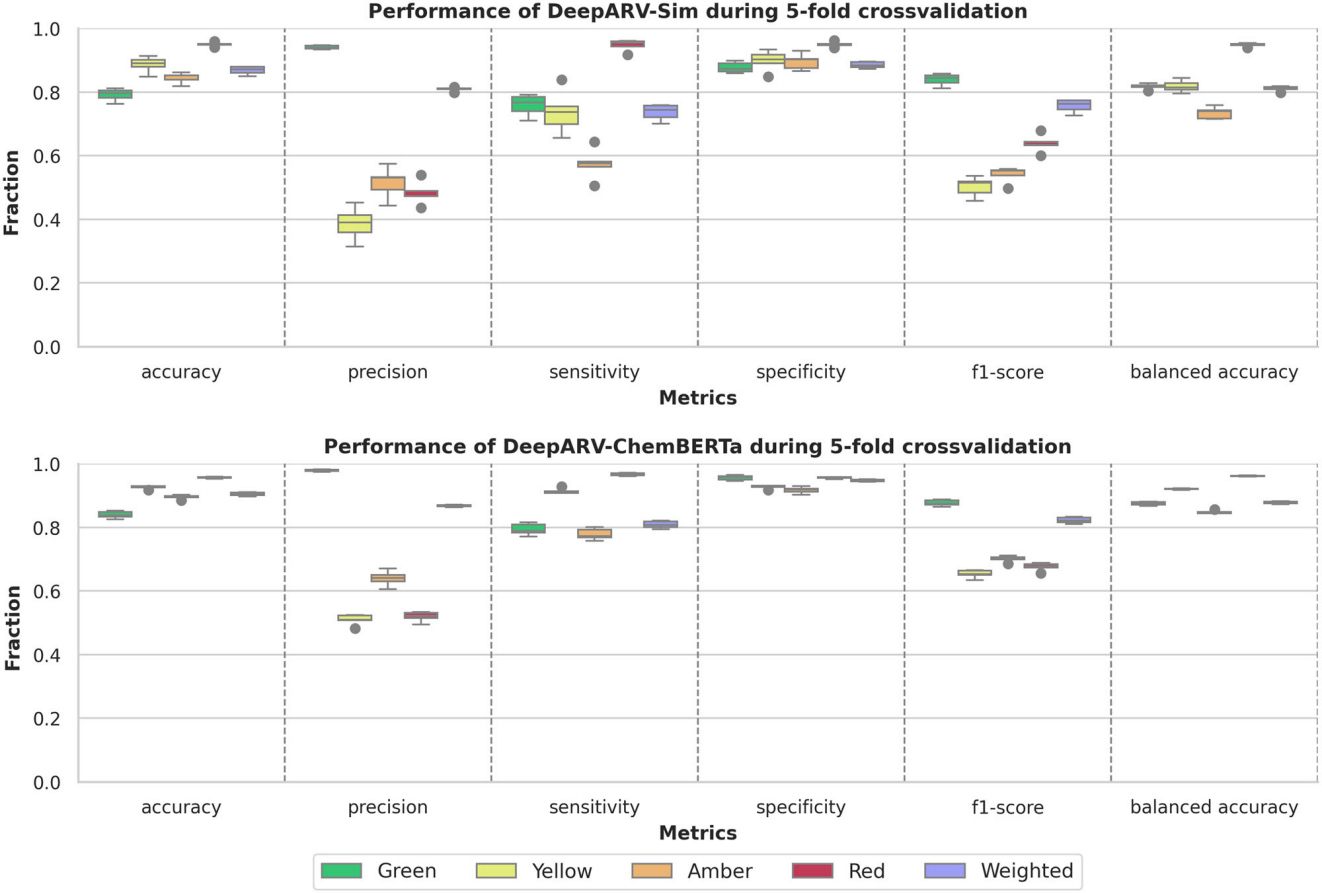
Performance of Single-Sim and Single-ChemBERTa on the independent test set. All trained models from 5-fold cross-validation were tested on the independent dataset with a total of 5103 DDIs, which was composed of 3221 Green, 358 Yellow, 1146 Amber and 378 Red. Single models share the same architecture as DeepARV-Sim and DeepARV-ChemBERTa, but without ensemble learning and class weight modification, namely Single-Sim and Single-ChemBERTa respectively. The box plot showed the distribution of the performance scores across accuracy, precision, sensitivity, specificity, f1-score and balanced accuracy of each DDI category and the weighted macros. Each box shows the quartiles of the result, with a line at the median. The whiskers extend to show the rest of the distribution, except for points that are determined to be outliners (displayed as dots) using a method that is a function of inter-quartile range.

Eight ARVs were randomly selected where associated drug pairs data containing either of those ARVs (*n* = 5103 pairs, 20% of the whole data) were kept blind from the 5-fold cross-validation to be used as an independent test set. On this independent dataset, the metrics for DeepARV-Sim were 0.703 ± 0.011 precision, 0.621 ± 0.032 sensitivity, 0.836 ± 0.018 specificity, 0.642 ± 0.029 f1-score and 0.729 ± 0.012 balanced accuracy. The metrics for DeepARV-ChemBERTa were 0.752 ± 0.012 precision, 0.675 ± 0.016 sensitivity, 0.878 ± 0.015 specificity, 0.7 ± 0.011 f1-score, and 0.776 ± 0.011 balanced accuracy. Evaluation metrics also included ROC curves with AUC measurements (Fig. 3). Both DeepARVs achieved the highest ROC-AUC values for the Red DDI category compared to other DDI categories, indicating a superior predictive ability in distinguishing the most clinically concerned DDI risk. The lowest values were observed for the Amber DDI category, with DeepARV-Sim exhibiting a lower ROC-AUC than DeepARV-ChemBERTa, implying a greater discriminative capacity for Amber risk with DeepARV-ChemBERTa.

**Fig. 4:**
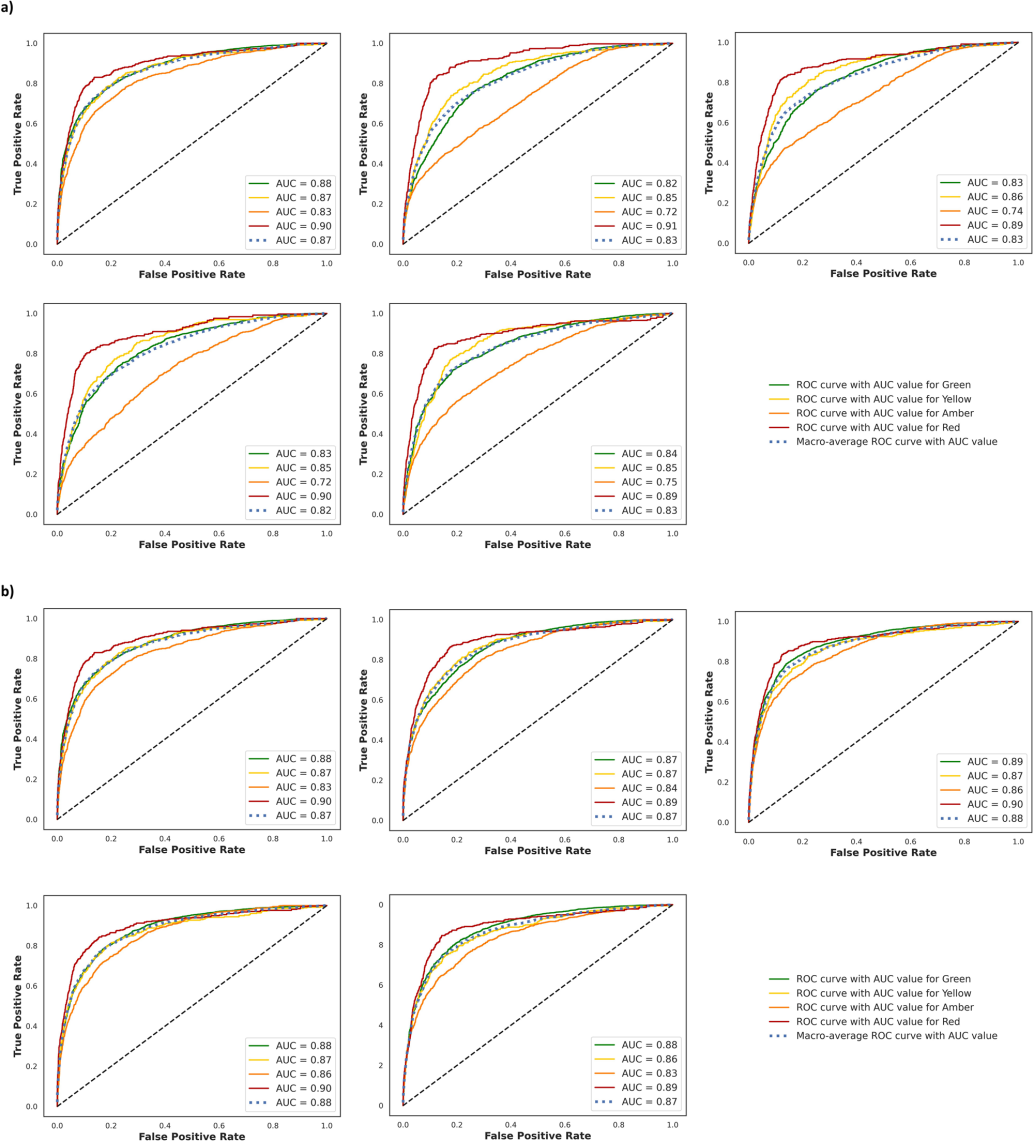
Receiver Operating Characteristics (ROC) Curve on the independent test set (*n* = 5103 DDI). **a** DeepARV-Sim and (**b**) DeepARV-ChemBERTa. Both models were tested multiple times on the independent dataset. The results showed ROC curves for each class as a plot of true positive rate (sensitivity) versus false positive rate (1-specificity) with AUC measurement.

**Fig. 5:**
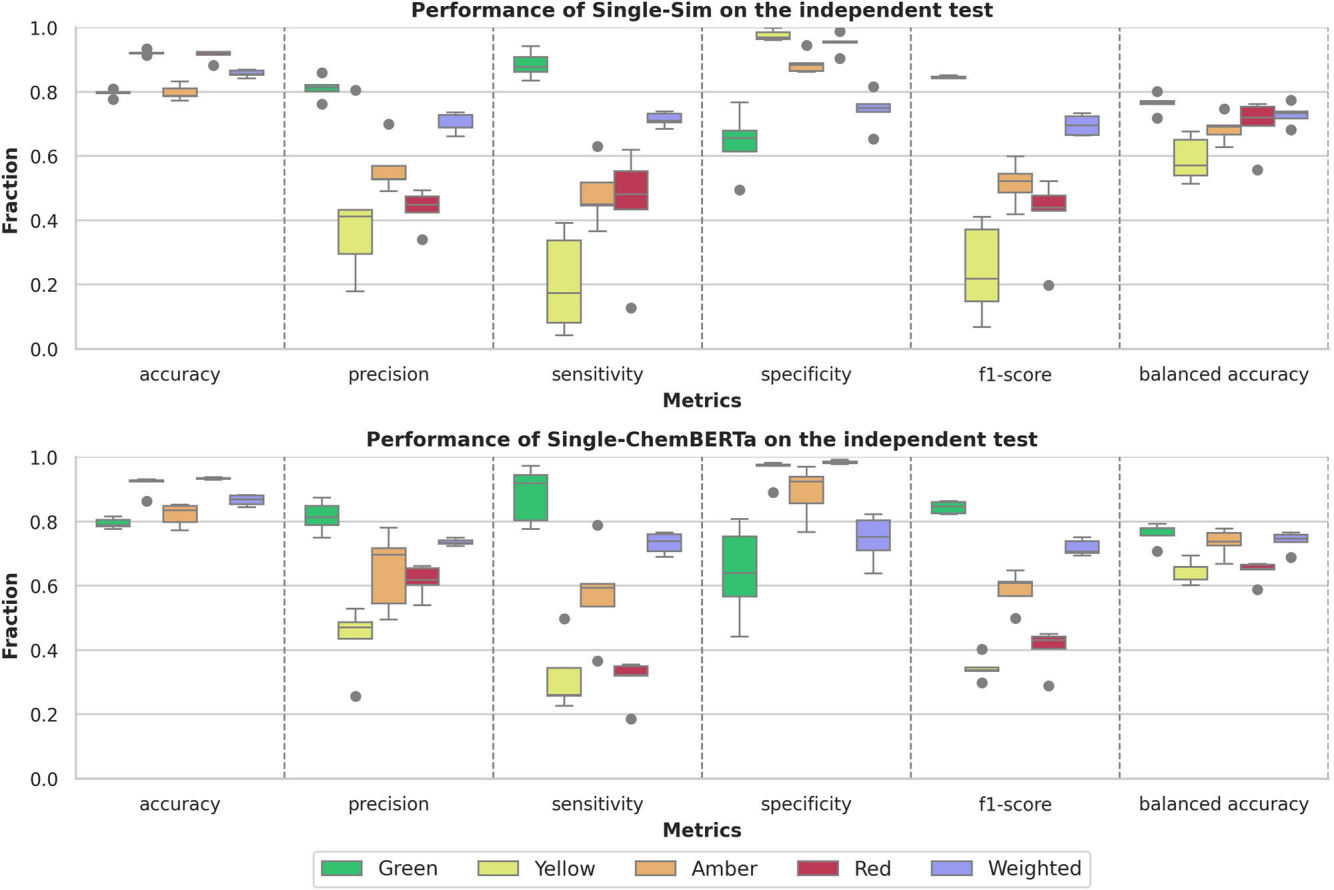
Performance of Single-Sim and Single-ChemBERTa on the independent test set. All trained models from 5-fold cross-validation were tested on the independent dataset with a total of 5103 DDIs, which was composed of 3221 Green, 358 Yellow, 1146 Amber and 378 Red. Single models share the same architecture as DeepARV-Sim and DeepARV-ChemBERTa, but without ensemble learning and class weight modification, namely Single-Sim and Single-ChemBERTa respectively. The box plot showed the distribution of the performance scores across accuracy, precision, sensitivity, specificity, f1-score and balanced accuracy of each DDI category and the weighted macros. Each box shows the quartiles of the result, with a line at the median. The whiskers extend to show the rest of the distribution, except for points that are determined to be outliners (displayed as dots) using a method that is a function of interquartile range.

To analyse the effectiveness of ensemble methods, the performance of the ensembles of DeepARV-Sim and DeepARV-ChemBERTa were compared against their single models, namely Single-Sim and Single-ChemBERTa respectively–both sharing the same architecture but without down-sampling techniques. The single models exhibited a higher weighted mean across all metrics but with a notably greater standard deviation, indicating a trade-off between performance and consistency (Fig. 4). A summary of the ground truth DDIs versus those predicted by both DeepARV-Sim, DeepARV-ChemBERTa and their corresponding single models (Single-Sim and Single-ChemBERTa) was represented as a normalised confusion matrix (Fig. 5). DeepARV-Sim displayed the highest predictive accuracy for the most critical Red DDI category, with a correct prediction rate of 0.71 ± 0.05, followed by DeepARV-ChemBERTa at 0.67 ± 0.04. In contrast, both single models misclassified approximately 50% of the Red DDI pairs. Additionally, DeepARV-ChemBERTa and Single-ChemBERTa exhibited a superior predictive ability for the Amber DDI category compared to DeepARV-Sim and Single-Sim.

### Comparison to other machine learning approaches

**Fig. 6:**
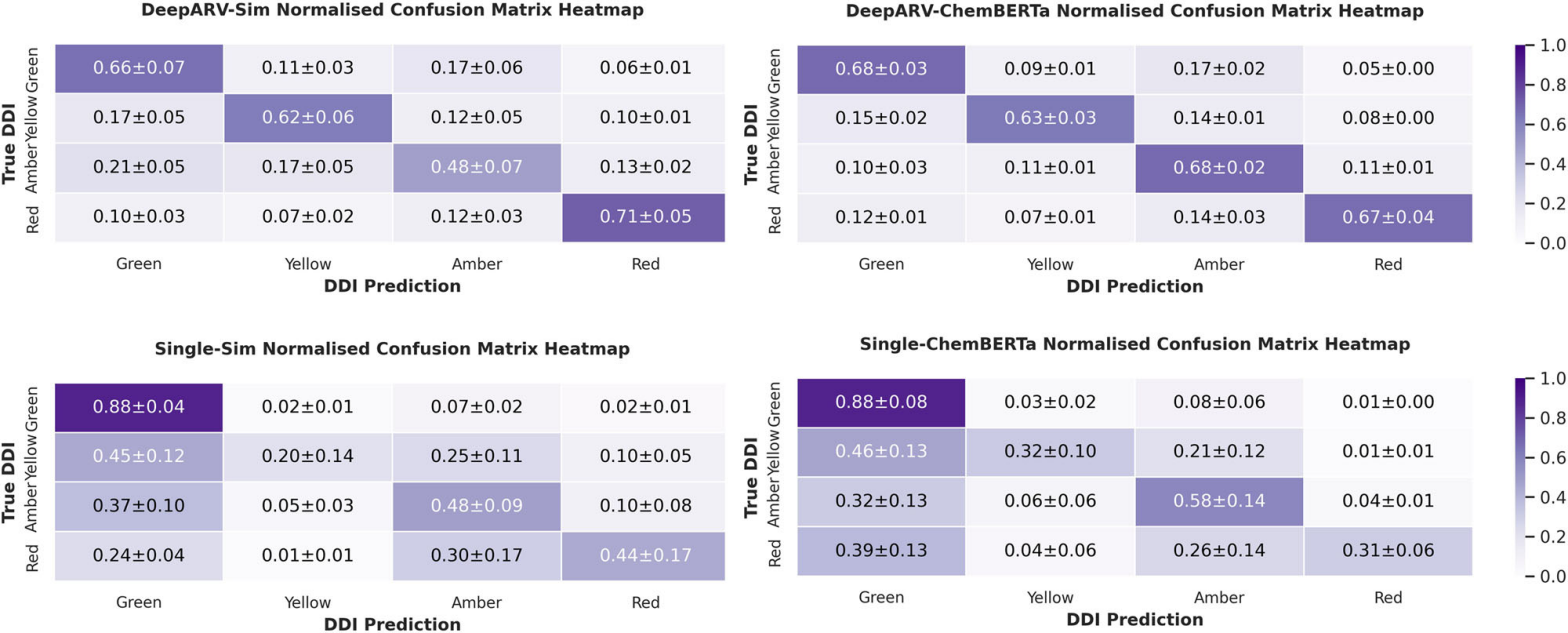
Normalised confusion matrix of DDI prediction by DeepARV-Sim, DeepARV-ChemBERTa, Single-Sim and Single-ChemBERTa versus the ground truth DDI. A total of 5103 DDIs composed of 3221 Green, 358 Yellow, 1146 Amber and 378 Red. Heatmap was applied to represent the concentration of values by colour depth.

To compare the performance of DeepARVs to other approaches, models including Gaussian Naïve Bayes (GaussianNB), Decision Trees (DTs) and Random Forest (RF) classifier were optimised and trained on the two datasets where features comprised of similarity profiles of drugs (suffixed as -Sim) and features constructed via ChemBERTa transformer (suffixed as -ChemBERTa). The performance of them on the independent test set was evaluated via precision, sensitivity, specificity, f1-score and balanced accuracy scores (Table 1). DeepARV-ChemBERTa outperformed other machine learning models across all metrics, yielding the highest score for precision (0.752), sensitivity (0.675), specificity (0.878), f1-score (0.695) and balanced accuracy (0.776). Normalised confusion matrix showed that GaussianNB-Sim had the most accurate prediction for the Red instances with a rate of 0.82 ± 0.01, followed by DeepARV-Sim with a rate of 0.71 ± 0.05 (Fig. 6).

**Table 1:** Performance of DeepARV and other machine learning approaches

	Precision	Sensitivity (Recall)	Specificity	F1-score	Balanced accuracy
GaussianNB-Sim	0.494	0.580	0.842	0.469	0.711
DFs-Sim	0.634	0.596	0.832	0.605	0.714
RF-Sim	0.680	0.656	0.860	0.666	0.758
DeepARV-Sim	0.703	0.621	0.836	0.642	0.729
GaussianNB-ChemBERTa	0.419	0.430	0.799	0.403	0.615
DFs-ChemBERTa	0.569	0.530	0.807	0.537	0.673
RF-ChemBERTa	0.744	0.550	0.798	0.591	0.674
DeepARV-ChemBERTa	**0.752**	**0.675**	**0.878**	**0.695**	**0.776**

Bold values present the highest performance for that metric across all the models.

## Discussion

**Fig. 7:**
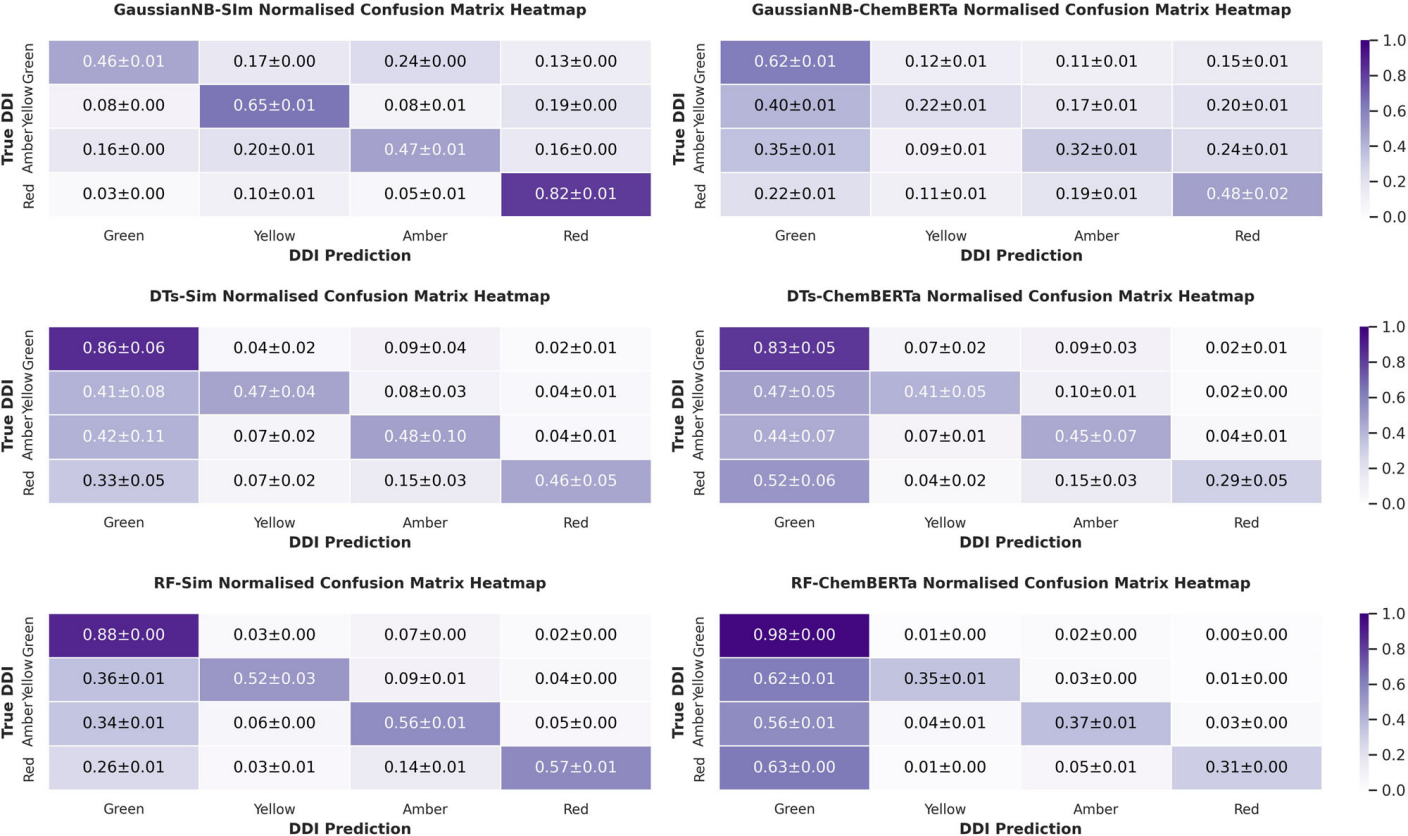
Performance of DeepARV-Sim and DeepARV-ChemBERTa on the independent test set. A total of 5103 DDIs composed of 3221 Green, 358 Yellow, 1146 Amber and 378 Red. Heatmap was applied to represent the concentration of values by colour depth.GaussianNB Gaussian Naïve Bayes, DTs Decision Trees, RF Random Forest, -Sim features were constructed as similarity profiles, -ChemBERTa: features were constructed as embeddings viaChemBERTa transformer-based model.

PLWH especially older individuals are more likely to present comorbidities and consequently are more likely to have polypharmacy leading to a higher risk of DDIs^29^. Therefore, there is a need for tools to predict risks associated with DDIs in order to prevent treatment related adverse events. We developed two DeepARV algorithms, namely DeepARV-Sim and DeepARV-ChemBERTa, to predict the risk of DDIs using structural molecular information of drugs. We evaluated the accuracy, sensitivity (recall), specificity, f1-score and balanced accuracy of both models against the University of Liverpool DDI HIV database (Fig. 7).

Skewed distribution across data, especially in the naturally occurring frequency of data (e.g., cancer detection with extreme disproportionate ratio of cancer and non-cancer patients) is one of the greatest concerns in developing a machine learning algorithm for real-world applications^30–32^. This is known as the imbalance problem, which is inherently difficult for the algorithm to capture the prediction of the minority class, potentially resulting in ignoring the entire class and thus reducing the relevance for future applications. Similarly, in our case, the Red DDI category was composed of the lowest number of pairs (5% of total drug pairs), however, the cost and consequences of making incorrect decisions against this minority class are much greater than other classes.

We evaluated the performance of both DeepARVs using the independent dataset, metrics of balanced accuracy was included to account for the skewed distribution of our DDI grading categories and as a trade-off between specificity and sensitivity^33,34^. DeepARV-ChemBERTa had a greater balanced accuracy score than DeepARV-Sim (0.776 ± 0.011 vs. 0.729 ± 0.012, respectively). However, DeepARV-Sim exhibited a superior predictive ability for the highest-risk DDI class, with a greater accurate prediction rate of 0.71 ± 0.05 compared to DeepARV-ChemBERTa with 0.67 ± 0.04.

The misclassification rate from the highest-risk Red DDI pairs to the no-risk Green DDI pairs is of the greatest concern clinically, measuring as 0.10 ± 0.03 for DeepARV-Sim and 0.12 ± 0.01 for DeepARV-ChemBERTa, with total of 378 Red DDI pairs. However, this misclassification rate for their corresponding single models were considerably greater, 0.24 ± 0.04 for Single-Sim and 0.30 ± 0.09 for Single-ChemBERTa, suggesting that DeepARVs outperformed their single counterparts in predicting minority but high-risk class instances. Moreover, both single models also exhibited a much greater variability and inconsistency in their performance across the test set and the evaluation iterations. These findings highlight the strength of both DeepARVs – ensemble models in addressing imbalanced data challenges.

On the other hand, among 3220 Green DDI pairs, approximately 23% of which were predicted as major-risk Amber/Red by both DeepARVs. Many of them were found to be supported by the guidelines from the European Medicine Agency, the National Institute for Health and Care Excellence UK (NICE) and Drugs.com (Table 2). One of such is DDI of lopinavir/ritonavir versus buprenorphine where both lopinavir and ritonavir may increase the concentration of buprenorphine, and thus monitoring and adjusting dose are recommended by the NICE^35,36^; another example is DDI of darunavir/cobicistat/emtricitabine/tenofovir alafenamide (DRV/c/FTC/TAF) versus clotrimazole where antifungals like clotrimazole inhibiting CYP3A may decrease the clearance of darunavir and cobicistat, and thus clinical monitoring is recommended by the European Medicines Agency^37^. Interestingly, Vivithanaporn et al.^38^ reported a significant inconsistency in severity grading across three major DDI databases – Drugs.com, Micromedex and the Liverpool HIV Interaction. A number of drug pairs between ARVs and antimicrobials were shown to have poor concordance in the DDI recognition, e.g., *minor* or *none* by Micromedex and Drugs.com but *major* (Amber/Red) by the Liverpool HIV Interaction database (e.g. DDI of atazanavir versus azithromycin, ciprofloxacin, mebendazole, primaquine, pentamidine and ribavirin). This disparity could be due to the low quality of evidence and/or lack of clinical data, complicating the evaluation of true accuracy of DeepARVs. In addition, it is important to highlight that our DeepARVs were developed based on the earlier Liverpool HIV Interaction database’s version (March 2021). Some of our novel DDIs are confirmed in the later version (January 2024), such as DDIs of efavirenz versus gastrointestinal agents including esomerprazole, lansoprazole, omeprazole and pantoprazole (Table 2). Our novel DDI predictions not only demonstrate the ability of our DeepARVs to predict previously undiscovered DDIs, but also emphasise the importance of cross-referencing multiple sources to enhance the robustness and accuracy of identified DDIs.

**Table 2:** Novel prediction by DeepARV-Sim and DeepARV-ChemBERTa

ARV – Comedication	DeepARV-Sim	DeepARV-ChemBERTa	Comedication Drug Class	Supported Evidence (NICE, Drugs.com)
Efavirenz – Esomeprazole*	X	X	*Gastrointestinal Agents*	Risk of altered drug metabolism.
Efavirenz – Lansoprazole*	X	X		
Efavirenz – Omeprazole*	X	X		
Efavirenz – Pantoprazole*	X			
Efavirenz – Oxcarbazepine	X	X		
Efavirenz – Ondansetron*		X		Risk of QT prolongation. Most manufacturers advise avoiding the use of two or more drugs that are associated with QT prolongation.
Efavirenz – Canagliflozin		X	*Anti-diabetics*	Risk of decreasing the exposure to the comedication. Manufacturer advises adjust Canagliflozin dose.
Efavirenz – Chlorpromazine*		X	*Antipsychotics, Neuroleptics*	Risk of QT prolongation. Most manufacturers advise avoiding the use of two or more drugs that are associated with QT prolongation.
Efavirenz – Formoterol*		X	*Bronchodilators*	Risk of hypokalaemia caused by the comedication (potentially increasing the risk of torsade de pointes) when taking with Efavirenz.
Efavirenz – Olodaterol		X		
Efavirenz – Furosemide*		X	*Hypertension, Heart Failure Agents*	Risk of hypokalaemia caused by the comedication (potentially increasing the risk of torsade de pointes) when taking with Efavirenz.
Efavirenz – Levonorgestrel (IUD)*		X	*Contraceptives, HRT*	Risk of decreasing the efficacy of the comedication
Lopinavir/Ritonavir – Buprenorphine	X	X	*Analgesics*	Risk of increasing the concentration of the comedication. Manufacturer advises to monitor and adjust the dose.
Lopinavir/Ritonavir – Doxycycline	X	X	*Antibacterials*	Risk of hepatotoxicity.
Lopinavir/Ritonavir – Miconazole		X	*Antifungals*	Risk of increasing the concentration of the ARVs. Manufacturer advises use with caution and adjusted dose.
Lopinavir/Ritonavir – Caspofungin		X		Risk of hepatotoxicity.
Lopinavir/Ritonavir – Dactinomycin	X	X	*Cancer Therapies*	Risk of hepatotoxicity.
Lopinavir/Ritonavir – Lenalidomide	X			
Lopinavir/Ritonavir – Oxandrolone	X	X	*Steroids*	Risk of hepatotoxicity.
Lopinavir/Ritonavir – Hydrocortisone*	X			Risk of increasing the exposure to the comedication. Manufacturer advises avoid or monitor adverse effects.
Lopinavir/Ritonavir – Thalidomide	X	X	*Other*	Risk of hepatotoxicity.
Lopinavir/Ritonavir – Acitretin	X			
Lopinavir/Ritonavir – Cabergoline*	X			Risk of increasing the concentration of comedication.
Lopinavir/Ritonavir – Almotriptan		X		Risk of increasing the exposure of the comedication.
Lopinavir/Ritonavir – Levonorgestrel (IUD)		X	*Contraceptives, HRT*	Risk of decreasing efficacy of the comedication.
Darunavir/Cobicistat/Emtricitabine/Tenofovir Alafenamide (DRV/c/FTC/TAF) – Acenocoumarol	X	X	*Anti-coagulant, Anti-platelet*	Risk of altered the anticoagulant effect of comedication. Manufacturer advises monitoring.
DRV/c/FTC/TAF – Clotrimazole	X	X	*Antifungals*	Risk of increasing the concentration of Darunavir and Cobicistat. Caution is warranted and clinical monitoring is recommended by the European Medicines Agency (EMA).
DRV/c/FTC/TAF – Almotriptan*	X		*Antimigraine Agents*	Risk of increasing the exposure to the comedication.
DRV/c/FTC/TAF – Cabergoline*	X	X	*Other*	Risk of increasing the exposure to the comedication.
DRV/c/FTC/TAF – Atovaquone	X			
DRV/c/FTC/TAF – Naloxone	X	X		Risk of increasing the concentration of the comedication. Dose adjustment may not be necessary but a careful clinical monitoring for signs of opiate toxicity is recommended by the EMA.
DRV/c/FTC/TAF – Hydrocortisone*	X		*Steroids*	Risk of increasing exposure to comedication. Manufacturer advises avoid or monitor adverse effects.
Raltegravir – Darunavir/Ritonavir (DRV/r)	X	X	*ARVs*	Risk of clinical rash, which was more commonly observed with regimens containing Raltegravir and Darunavir compared to those containing Raltegravir without darunavir or darunavir without Raltegravir (EMA).

*These DDIs have been re-classified and updated on the Liverpool HIV Drug Interaction database, https://www.hiv-druginteractions.org (Last review on January 12, 2024).

Both DeepARV-Sim and DeepARV-ChemBERTa also showcased their potential in discovering novel interactions previously overlooked by the existing system. It should be noted that certain novel DDIs were exclusively detected by DeepARV-Sim such as efavirenz – omeprazole, and lopinavir/ritonavir – hydrocortisone, while others were identified solely by DeepARV-ChemBERTa such as efavirenz – canagliflozin, and lopinavir/ritonavir – levonorgestrel (IUD). Therefore, integrating the synergistic strengths of DeepARV-Sim and DeepARV-ChemBERTa into a unified ensemble model presents a promising avenue for future work. Moreover, data split for ensemble models could be functionally informed so that each model would be able to establish a complementary strength for the prediction task.

The Liverpool HIV Interaction database undergoes continuous updates, with new therapies added and existing drug pairs re-scaled (downgraded/upgraded) based on emerging evidence from clinical DDI studies, case reports or clinical practice. Our DeepARV models represent a promising tool for enhancing the screening process of the database. Given the inherent challenge and time-consuming nature of identifying DDI risk, DeepARVs offer a powerful means to examine the clinical DDI pattern associated with molecular structure in a time- and cost- efficient manner. This capability of DeepARVs provides an additional perspective and criteria during the development/maintenance of ARV DDI databases and the drug development process.

As a limitation of our study, exploring the performance of DeepARVs against the DrugBank Open Data or other latest state-of-the-art models presented challenges due to variations in DDI classification systems. The University of Liverpool HIV Interaction specialises in determining clinically relevant DDIs of HIV medicine, its grading system ranks the clinical significance of an interaction from ‘no interaction’ (green flag), ‘interaction of weak intensity not requiring additional action’ (yellow flag), ‘potentially clinically relevant DDI requiring either dose adjusting or close clinical monitoring’ (amber flag), to ‘contraindicated’ (red flag). On the other hand, the DrugBank Open Data (version 5.0) provides descriptive DDI information outlining the mechanism or consequences of the interaction. For example, DrugBank describes the interaction between efavirenz and terbinafine as ‘The metabolism of Terbinafine can be decreased when combined with Efavirenz’ (https://go.drugbank.com/drugs/DB00625), whereas the Liverpool HIV Interaction classifies it as ‘yellow’ indicating potential interaction of weak clinical relevance for which additional action/monitoring or dosage adjustment is not required. For future work, further refining the architecture of DeepARVs to further investigate its performance on a standardised DDI classification system should be considered to enable the benchmarking of state-of-the-art-models, and to enhance the applicability and robustness of DeepARVs.

DeepARV-Sim and DeepARV-ChemBERTa were developed exclusively integrating structural data, into an automated analytical model, providing an evaluation of ARV DDI risks. The performance of DeepARVs were comprehensively evaluated and could generate opportunities for similar applications for other disease areas. DeepARVs define innovative opportunities for an integrated application of AI approaches in the drug development process for the rational prediction of risk related to DDIs.

## Methods

### University of Liverpool Drug Interaction

The University of Liverpool Drug Interaction provided the DDI identification between ARVs and comedication (https://www.hiv-druginteractions.org/). An overview of the database (March 2021 version) is outlined in Supplementary Table 2. The assessment of the DDI potential for a given drug combination is evaluated following a systematic approach which consists first to compile the pharmacokinetic and pharmacodynamic characteristics of the co-administered drugs including also available in vitro or clinical DDI studies or case reports. The risk of having a clinically relevant DDI is typically low if the co-administered drugs have different elimination pathways or if the change in exposure is unlikely to be of clinical relevance or lead to safety concern. If there is evidence that a DDI may occur, the clinical relevance of the DDI is subsequently evaluated by taking into account factors such as the magnitude of the pharmacokinetic change, the therapeutic index of the drug or the possibility to monitor the drug. The recommendation of the product label is also taken into consideration when classifying the DDI into a risk category (https://www.hiv-druginteractions.org/site_updates). There are four categories for the DDI risk: 1) Green – No clinically significant interaction expected. 2) Yellow – Potential interaction of weak clinical relevance for which additional action/monitoring or dosage adjustment is not required. 3) Amber – Potential clinically relevant interaction that can be managed by clinical monitoring, alteration of drug dosage or timing of administration. 4) Red – These drugs should not be co-administered as they may cause a deleterious effect (e.g., loss of efficacy or toxicity of the ARV drug or co-administered drug).

### DeepARV-similarity

#### Morgan fingerprint

The molecular structure of each drug in SMILES format was extracted from PubChem Substances and Compound database (version updated in March 2019) via *PubChemPy*. Chemical fingerprints of each drug were then calculated using Morgan fingerprints with *RDKit* (version 2021.09.3), where neighbours of each atom up to a radius of 2 were recorded as binary numerical format (0 or 1) in 1024 bits. For combination therapy, the final fingerprint was determined by concatenating bits of ‘1’ from corresponding drugs while maintaining the fingerprint size.

#### Structural similarity profile

Similarity in structure between two drugs was measured by the Tanimoto coefficient based on the industry standard^39^, where the intersection of common chemical fingerprints is divided by the union of fingerprints of the two drugs (Supplementary Fig. 2). Tanimoto coefficient ranges between 0 and 1, reflecting the degree of structural similarity between two drugs being compared, where a higher value indicates higher similarity. To construct a similarity profile, each drug was compared to a fixed reference drug list from the database (*n* = 688). This approach was applied based on the assumption that similar drugs are likely to have similar interactions. Given a drug pair of interest, structural similarity profiles of both drugs were concatenated and fed into the input layer of the neural network, which was optimised for predicting DDI. This approach was referred to as DeepARV-Sim.

### DeepARV-ChemBERTa

#### Transformer-based model

ChemBERTa, developed by Chithrananda et al. ^13^, is a variant of BERT transformer-based model that learns molecular structures through semi-supervised pretraining of the language model. ChemBERTa was pretrained on 10 million SMILES from PubChem using the same procedure used by RoBERTa. It involved masking 15% of tokens in each SMILES string and assigning a maximum sequence length of 256 characters. Through the use of the byte pair encoding (BPE) algorithm, ChemBERTa has learnt to predict masked tokens consisting of multiple atoms and functional groups.

#### Feature extractor and fine-tuning

ChemBERTa was downloaded from seyonec/PubChem10M_SMILES_BPE_450k tokenizer via Huggingface: https://huggingface.co/seyonec/PubChem10M_SMILES_BPE_450k/tree/main. The final hidden layer was adapted to serve as a featuriser that outputted embeddings of length of 768 bits for a given drug SMILES. The embeddings were concatenated for corresponding drug pairs as input feature to the neural network, which was subsequently fine-tuned for the prediction of DDIs, called DeepARV-ChemBERTa.

### 5-fold stratified cross-validation

DeepARVs were trained to classify four types of DDI (Green, Yellow, Amber, and Red) between ARVs and comedications. The training set of 25,039 drug pairs (80% of the whole data) were split for a stratified 5-fold cross validation, maintaining the distribution of DDI classes in each validation step (Supplementary Table 3). 4-Fold data was used for training the model and the remaining fold was used for validation. This process repeated five times, with each fold serving in the validation step once.

### Sampling techniques and ensemble models

As the dataset had a skewed distribution of pairs across four severity grading classes, under-sampling techniques with ensemble methods and class weight balance were employed. Undersampling technique was applied to the training dataset by considering Green DDI category as a majority class, associated drug pairs (*n* = 13,811) were reduced to 5 subsets (n = 2762 per each set); pairs from remaining DDI categories were kept the same within the subsets (Table 3).

**Table 3:** Undersampling the majority class – Green DDI category during 5-fold cross-validation process

DDI class	Before sampling		Sampling to five subsets	
	Drug pairs	Distribution	Drug pairs per subset	Distribution
**Green**	13,811	72%	2762	36.3%
**Yellow**	1472	7.6%	1472	17.2%
**Amber**	3052	16%	3052	36.1%
**Red**	895	4.4%	895	10.4%
**Total**	19,230	100%	8181	100%

Five ensemble models were built with the same neural network architecture where each model was trained on each subset. Class weight balance technique was applied to all ensemble models based on Eq. (1) where the final weight of each DDI class was inversely proportionate to the number of samples within that class.

1 $${weight}\,{of}\,{each}\,{class}=\frac{{total}\,{number}\,{of}\,{samples}}{{total}\,{number}\,{of}\,{classes}\times {number}\,{of}\,{samples}\,{within}\,{that}\,{class}}$$

DDI predictions from the five ensemble models were aggregated where each ensemble model predicted probabilities across DDI classes. Soft voting technique was applied to determine the final prediction by averaging the predicted probabilities and selecting the DDI class with the highest probability.

### Independent test

To construct the independent test, eight ARVs were randomly selected to be excluded from training and used for testing. Five ARV drug classes available on the Liverpool HIV Drug Interaction database (version March 2021) included: 1) Entry and Attachment Inhibitors, 2) Integrase Inhibitors (INSTIs), 3) Non-Nucleoside Transcriptase Inhibitors (NNRTIs), 4) Nucleoside/tide Analogues (NRTIs/NtRTIs), 5) Protease Inhibitors (PIs). An overview of chemical structure of single ARVs and related drug classes was shown in Supplementary Fig. 3. ARVs within the same class often share common structural similarities, however, variation in chemical structures within the same class persists and is of great significance to optimise the therapeutical efficacy and overcome viral resistance. The structural differences arise the most across ARV drug classes due to their distinct mechanisms of action and/or targets. Therefore, the selection of test set was comprised of five single ARVs and three combination ARVs, ensuring a comprehensive representation across all classes of ARV to inspire the confidence of the prediction: 1) Emtricitabine (FTC) - NRTIs/NtRTIs, 2) Efavirenz (EFV) - NNRTIs, 3) Darunavir/Cobicistat/Emtricitabine/Tenofovir alafenamide (DRV/c/FTC/TAF) - PIs, 4) Lopinavir/Ritonavir (LPV/r) - PIs, 5) Doravirine/Lamivudine/Tenofovir-DF (DOR/3TC/TDF) - NNRTIs, 6) Maraviroc (MVC) – Entry Inhibitors, 7) Tipranavir (TPV) - PIs, and, 8) Raltegravir (RAL) - INSTIs. The performance of the model was evaluated on this set with a total of 5,103 drug pairs where Green was composed of 3221 pairs, Yellow of 358 pairs, Amber of 1146 pairs and Red of 378 pairs.

### Optimisation of the neural network

The architecture of both DeepARVs was a neural network. To optimise DeepARVs, a number of hidden layers of {1, 2, 3, 4, 5} and a number of neurons in hidden layers of {2048, 1024, 512, 256, 128} were tested. In the model, the activation function of hidden layers was tested using either Rectified linear activation function (ReLU) following Eq. (2) or Tanh following Eq. (3). Dropout rate of {0.2, 0.4} was tested.

2 $$f\left(x\right)=\left\{x,0\right\}$$

3 $$tanh\, \left(x\right)=\frac{{e}^{2x}-1}{{e}^{2x}+1}$$

The optimised architecture of DeepARV-Sim was composed of four hidden layers with {1024, 512, 256, 128} number of neurons respectively, the activation function of hidden layers was ‘ReLu’ and of the output layer was ‘softmax’, dropout rate was {0.2} and learning rate was set to {0.001} (Supplementary Fig. 4).

The optimised architecture of DeepARV-ChemBERTa was composed of two hidden layers with {256, 128} number of neurons respectively, the activation of hidden layers was ‘Tanh’ and of the output layer was ‘softmax’, dropout rate was {0.2} and learning rate was set to {0.001} (Supplementary Fig. 5).

The cost function used for training was Sparse Categorical Crossentropy, also known as Softmax Loss, which evaluates crossentropy between the predicted probability distribution and the true labels. Training Adaptive Moment Estimation (Adam) optimiser (beta_1 = 0.9, beta_2 = 0.999, epsilon = 1e-07) was employed to minimise the cost function. Adam uses momentum and adaptive learning rates to converge efficiently. To prevent overfitting, early stopping was applied as a callback during training.

### Other machine learning approaches for benchmarking

Gaussian Naïve Bayes (GaussianNB), Decision Trees (DTs), Random Forest (RF) were built using scikit-learn (version 1.0.2; https://scikit-learn.org/) with ‘GridSearhCV’ optimising function. Hyperparameter tuning was performed for both features of similarity profile via molecular fingerprints-Tanimoto score and embeddings via ChemBERTa, the optimal model was suffixed as -Sim and -ChemBERTa respectively.

#### Gaussian naïve bayes

GaussianNB is a variant of Naïve Bayes, which is a supervised learning algorithm that supports continuous values and assumes normal distribution across all classes. GaussianNB has been well-known for its effectiveness and efficiency in multiclass prediction^40^. The likelihood of features is assumed to be Gaussian where ${\sigma }_{y}$ and ${\mu }_{y}$ are estimated using maximum likelihood (4).

4 $$P\left(y\right)=\frac{1}{\sqrt{2\pi {\sigma }_{y}^{2}}}exp \,exp \left(-\frac{{({x}_{i}-{\mu }_{y})}^{2}}{2{\sigma }_{y}^{2}}\right)$$

Hyperparameter tuning for GaussianNB was performed on two parameters: ‘priors’ – representing the prior probability of a class, and ‘var_smoothing’ – a stability calculation to account for variance of the features. ‘Priors’ were tested on two sets of (0.72, 0.076, 0.16, 0.044), which was corresponding to Green, Yellow, Amber, Red classes, and of (0.1, 0.1, 0.3, 0.5), where the highest importance was assigned to the Amber and Red classes. The testing range for ‘var_smoothing’ was {1e-9, 1e-8, 1e-7}.

The best parameters for GaussianNB-Sim were {‘priors’ = (0.72, 0.076, 0.16, 0.044), ‘var_smoothing’ = 1e-07}, and for GaussianNB-ChemBERTa were {‘priors’ = (0.72, 0.076, 0.16, 0.044), ‘var_smoothing’ = 1e-09}.

#### Decision trees

DTs are a non-parametric supervised learning algorithm and is a tree-like model. It is composed of a hierarchical tree root with internal nodes representing a feature, branches representing a decision rule and leaf nodes, where each of them is the decision or the outcome. The prediction of a target value is achieved by learning simple decision rules inferred from the feature. DTs are widely used due to their simplicity, versatility, and interpretability^41^.

The grid search to explore hyperparameters for DTs included: i) the maximum depth (‘max_depth’) in the range of {None, 5, 10, 20}, ii) the minimum number of samples required to split an internal node (‘min_samples_split’) with options of {2, 5, 10}, iii) the minimum number of samples required to be in a leaf node (‘min_samples_leaf’) over a range of {1, 2, 4}, iv) the maximum features considered for splitting a node (‘max_features’) with choices of {‘sqrt’, ‘log2’}, and, v) the function to measure the quality of a split, either ‘gini’ for Gini impurity (5) or ‘entropy’ for information gain (6) was explored as follows:

5 $${Gini}\left(p\right)=1-\mathop{\sum }\limits_{i=1}^{c}{({p}_{i})}^{2}$$

Where ${Gini}\left(p\right)$ is the Gini impurity for a particular node, $c$ is the number of classes (*n* = 4), ${p}_{i}$ is the probability of belonging to class $i$ in a given node. The Gini impurity is minimised when all samples in a node belong to the same class (pure node).

6 $${Entropy}\left(p\right)=-\mathop{\sum }\limits_{i=1}^{c}\,{p}_{i}({p}_{i})$$

Where ${Entropy}(p)$ is the entropy for a particular node, $c$ is the number of classes (*n* = 4), ${p}_{i}$ is the probability of belonging to class $i$ in a given node. Similar to the Gini impurity, Entropy is minimised when all samples in a node belong to the same class.

The best parameters for DTs-Sim were {‘criterion’ = ‘gini’, ‘max_depth’ = 20, ‘max_features’ = ‘sqrt’, ‘min_samples_leaf’ = 1, ‘min_samples_split’ = 2}, and for DTs-ChemBERTa were {‘criterion’ = ‘gini’, ‘max_depth’ = 20, ‘max_features’ = ‘sqrt’, ‘min_samples_leaf’ = 1, ‘min_samples_split’ = 10}.

#### Random forest

RF is an ensemble learning method of decision trees that builds multiple trees independently during training and the final prediction is determined by a majority vote among the individual trees^42^. RF presents advantages over a single decision tree such as a lower risk of overfitting due to its ensemble approach, and robustness to noisy data achieved via a voting technique.

Optimising RF hyperparameters included: i) the number of decision trees in the forest, (n_estimators) tested over a range of {100, 200, 300}, ii) the maximum depth of trees that helps to control overfitting, (‘max_depth’) of choices {None, 5, 10, 20}, iii) the minimum number of samples required to split an internal node that have impact on the tree granularity and generalisation, (‘min_samples_split’) with options {2, 5, 10}, iv) the minimum number of samples required to form a leaf node (‘min_samples_leaf’) tested over values {1, 2, 4}, v) the maximum number of features for splitting at each node, allowing randomness and diversity among trees, (‘max_features’) with either choice of square root (‘sqrt’) or base-2 logarithm (log2), and vi) options of whether using bootstrap sampling technique during the generation of individual trees (‘bootstrap’).

The best parameters for RF-Sim were {‘n_estimators’ = 100, ‘max_depth’ = 20, ‘min_samples_split’ = 2, ‘min_samples_leaf’ = 1, ‘max_features’ = ‘sqrt’, ‘bootstrap’ = False}, and for RF-ChemBERTa were {‘n_estimators’ = 100, ‘max_depth’ = 20, ‘min_samples_split’ = 5, ‘min_samples_leaf’ = 1, ‘max_features’ = ‘sqrt’, ‘bootstrap’ = False}.

### Evaluation criteria

Evaluation metrics included accuracy, precision, sensitivity (also known as recall), specificity, f1-score (7), balanced accuracy (8) and ROC-AUC score. Positive and negative classifications were defined as a binary classification for every class individually e.g. for Green DDI category: True Positive (TP) instances constitute true target Green class predicted as Green; True Negative (TN) instances include target class of Yellow, Amber or Red not predicted as Green; False Negative (FN) instances are target Green class predicted as either Yellow, Amber or Red; False Positive (FP) instances are target class of Yellow, Amber or Red predicted as Green.

7 $$F1=\frac{2\times {precision}\times {recall}}{{precision}+{recall}}=\frac{2\times {TP}}{2\times {TP}+{FP}+{FN}}$$

8 $${Balanced}\,{accuracy}=\frac{{sensitivity}+{specificity}}{2}$$

The overall performance of the model was computed using weighted macro-average. It combines the macro-average metric (e.g. accuracy, precision, sensitivity) (9) and weighting based on class frequencies (10) to account for class imbalance. To calculate weighted macro-average, the macro-average metric was weighted by the corresponding class’s frequency and summed up (11).

9 $${Averaged}\,{Macro}\,{Metric}=\frac{1}{N}\mathop{\sum }\limits_{i=1}^{N}{{Metric}}_{i}$$

10 $${{Weight}}_{i}=\frac{{Number}\,{of}\,{instances}\,{in}\,{class}\,i}{{Total}\,{number}\,{of}\,{instances}}$$

11 $${Weighted}\,{Averaged}\,{Macro}=\mathop{\sum }\limits_{i=1}^{N}{{Weight}}_{i}\times {{Metric}}_{i}$$

Where $N$ is the total number of classes, in our case, $N=4$.

### Overall scheme (Fig. 1)

At the data-level, instead of randomly removing drug pairs with redundancy, our pipeline employed a combination of undersampling and ensemble techniques to avoid the removal of any samples. Briefly, the under-sampling method was applied to reduce the size of the majority class–Green to five equal subsets while keeping all the samples from the minority classes–Amber, Yellow and Red within each subset. The ensemble methods involved the construction of five neural network models with the same architecture, each model was trained on each subset. As a result, the uneven frequency of {Green: Amber: Yellow: Red} with a ratio of approximately {30: 7: 3: 2} respectively was minimised to {7:7:3:2} respectively.

At the algorithm-level, the weight for each class prediction is algorithmically calculated to be equal by assuming normal distribution of the data. In our case, all the ensemble models of DeepARVs were modified to examine the skewed distribution across DDI classes, where the weight for each class was inversely proportional to the frequency of that class. As a result, class weights in descending order of 2.429, 1.448, 0.692, and 0.688 were applied to the DDI class of Red, Yellow, Amber, and Green, respectively. During the learning process, the penalties for a misclassification attempt was calculated through class weight, called the cost function. By giving the highest weight to the Red DDI category, the incorrect prediction of this instance was penalised the most, increasing the cost of this class and so its importance. The ultimate purpose was to decrease the likelihood of misclassification against the Red DDI pairs and to reduce the bias towards the majority class-Green for future predictions.

The predicted class probabilities by ensemble models were pooled and averaged, the DDI class with the highest probability was the final prediction.

### Reporting summary

Further information on research design is available in the Nature Research Reporting Summary linked to this article.

### Supplementary information

Supplementary Information

Reporting summary

## Data Availability

The datasets used and/or analysed during the current study available from the corresponding author on reasonable request. DDI risks between ARVs and comedication are publicly available at the Liverpool HIV Interaction Checker, https://www.hiv-druginteractions.org/. Molecular structures are available at PubChem Substance and Compound databases, https://pubchem.ncbi.nlm.nih.gov/.

## Code availability

The underlying code for this study is available in GitHub and can be accessed via this link https://github.com/ThaoPham96/DeepARV.
