# Supplementary material for: DeepARV: ensemble deep learning to predict drug-drug interaction of clinical relevance with antiretroviral therapy
Source: NPJ Syst Biol Appl. 2024 May 6;10:48. doi: 10.1038/s41540-024-00374-0 (PMC11074332; doi:10.1038/s41540-024-00374-0)
Supplement: Supplementary file 1 — Supplementary Information [file 41540_2024_374_MOESM1_ESM.pdf]

Supplementary Information for

DeepARV: Ensemble Deep Learning to Predict Drug-Drug Interaction of Clinical Relevance with Antiretroviral Therapy

Molecular structural similarity analysis

As an example, five anticonvulsant agents were pooled for comparison, where a reference compound was phenobarbitone, and test compounds included phenytoin, primidone, ethosuximide and topiramate. To illustrate DDI pattern between ARVs and these agents, colour coding of 4 DDI risks were derived from the Liverpool DDI HIV database as: 1) Green – No clinically significant interaction expected. 2) Yellow – Potential interaction of weak clinical relevance for which additional action/monitoring or dosage adjustment is not required. 3) Amber – Potential clinically relevant interaction that can be managed by clinical monitoring, alteration of drug dosage or timing of administration. 4) Red – These drugs should not be co-administered as they may cause a deleterious effect (e.g., loss of efficacy or toxicity of the ARV drug or coadministered drug). Structural similarity Tanimoto scores of phenobarbitone versus primidone (0.68) and phenytoin (0.52) were higher than which versus ethosuximide (0.28) and topiramate (0.07) (**Supplementary Table 1**). High structural similarity Tanimoto score of phenobarbitone versus primidone and phenytoin and given that all have inducing properties of pharmacokinetic interactions, contributing to consistent DDI patterns against ARVs. On the other hand, ethosuximide and topiramate have low Tanimoto score versus phenobarbitone and are not inducers of drug metabolising enzymes, therefore resulting in distinguished DDI patterns.

Supplementary Table 1. Example of DDI pattern between ARVs and comedications from the anticonvulsant class. Considering phenobarbitone as the reference drug, query drugs with Tanimoto score are primidone (0.68), phenytoin (0.52), ethosuximide (0.28) and topiramate (0.07). G (Green), Y (Yellow), A (Amber), R (Red).

**ARVs**	**Phenobarbi-tone (reference)**	**Primidone (0.68)**	**Phenytoin (0.52)**	**Ethosuxim-ide (0.28)**	**Topiramate (0.07)**
Enfuvirtide (ENF, T20)	G	G	G	G	G
Maraviroc (MVC)	A	A	A	G	G
Dolutegravir/Rilpivirine (DTG/RPV)	R	R	R	G	G
Dolutegravir/Abacavir/Lamivudine (DTG/ABC/3TC)	A	A	A	G	G
Bictegravir/ Emtricitabine/Tenofovir alafenamide (BIC/FTC/TAF)	R	R	R	G	G
Elvitegravir/Cobicistat/Emtricitabine/Tenofovir alafenamide (EVG/c/FTC/TAF)	R	R	R	A	G
Dolutegravir/Lamivudine (DTG/3TC)	A	A	A	G	G
Elvitegravir/Cobicistat/Emtricitabine/Tenofovir-DF (EVG/c/FTC/TDF)	R	R	R	A	A
Cabotegravir (CAB)	R	R	R	G	G

Dolutegravir (DTG)	A	A	A	G	G
Raltegravir (RAL)	A	A	A	G	G
Doravirine (DOR)	R	R	R	G	G
Etravirine (ETR)	R	R	R	A	G
Dapivirine (DPV, PrEP)	G	G	G	G	G
Rilpivirine (RPV)	R	R	R	G	G
Nevirapine (NVP)	R	R	A	A	G
Rilpivirine/Emtricitabine/Tenofovir alafenamide (RPV/FTC/TAF)	R	R	R	G	G
Efavirenz (EFV)	A	A	A	A	G
Doravirine/Lamivudine/ Tenofovir-DF (DOR/3TC/TDF)	R	R	R	G	A
Emtricitabine/Tenofovir alafenamide (FTC/TAF, PrEP)	R	R	R	G	G
Zidovudine (AZT, ZDV)	A	A	Y	A	G
Stavudine (d4T)	G	G	G	G	G
Emtricitabine/Tenofovir-DF (FTC/TDF, PrEP)	G	G	G	G	A
Emtricitabine (FTC)	G	G	G	G	G
Tenofovir-DF (TDF)	G	G	G	G	A

Abacavir (ABC)	Y	Y	Y	G	G
Fosamprenavir (FPV)	A	A	A	A	G
Darunavir/Cobicistat/Emtricitabine/Tenofovir alafenamide (DRV/c/FTC/TAF)	R	R	R	A	G
Tipranavir (TPV)	A	A	A	A	G
Indinavir (IDV)	A	A	A	A	A
Darunavir/cobicistat (DRV/c)	R	R	R	A	G

University of Liverpool Drug Interaction Database

A brief overview of the University of Liverpool DDI HIV database was presented in **Supplementary Table 2**. Colour coding of 4 DDI types as: 1) Green – No clinically significant interaction expected. 2) Yellow – Potential interaction of weak clinical relevance for which additional action/monitoring or dosage adjustment is not required. 3) Amber – Potential clinically relevant interaction that can be managed by clinical monitoring, alteration of drug dosage or timing of administration. 4) Red – These drugs should not be co-administered as they may cause a deleterious effect (e.g., loss of efficacy or toxicity of the ARV drug or co-administered drug).

Supplementary Table 2. Brief summary of the Liverpool HIV database (as per March 2021).

**DDI types**	**DDI pairs (n = 30,142)**
Green	20,485
Yellow	2,182
Amber	4,962
Red	1,497
**ARV class**	**ARV drugs (including combination, n = 42)**
Protease inhibitors	11
Integrase inhibitors	10
Nucleoside/tide analogues	9
Non-nucleoside reverse transcriptase inhibitors	8

Entry and attachment inhibitors	4
**Example of comedication class**	**Comedication (n = 695)**
Hypertension/Heart failure agents	55
Antibacterial	52
Cancer therapies	52
Antidepressants	28
Herbals/Supplements/Vitamins	26
Antivirals	26

5-fold stratified cross-validation

The training set of 24,039 drug pairs (80% of the whole data) were split for a stratified 5-fold cross validation, maintaining the distribution of DDI classes in each validation step (**Supplementary Table 3**).

Supplementary Table 3. 5-fold stratified cross-validation dataset split.

DDI class	5-fold cross-validation dataset (n = 24,039)	4-fold training (n = 19,230)	1-fold validation (n = 4,809)
Green	17,264	13,811	3,453
Yellow	1,840	1,472	368
Amber	3,816	3,052	763
Red	1,119	895	224

DeepARV accurately predicts DDI classes

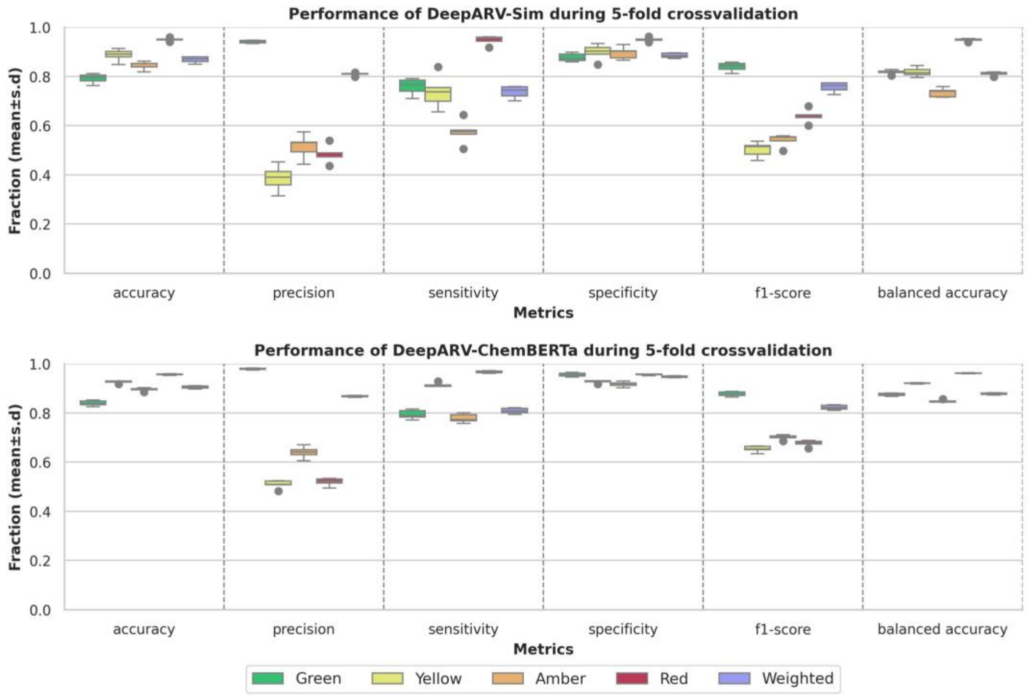

Supplementary Figure 1. Performance of DeepARV-Sim and DeepARV-ChemBERTa during 5-fold cross-validation. The process involved training and evaluating five times on a dataset of 25,039 drug pairs. For each iteration, 4-fold of data (n = 19,230) was applied for training and the remaining 1-fold (n = 5,809) for evaluation. Performance metrics for each iteration included accuracy, precision, sensitivity, specificity, f1-score and balanced accuracy for each DDI class and the weighted macros. The box plot showed the distribution of the performance metrics across all five iterations. Each box shows the quartiles of the result, with a line at the median. The whiskers extend to show the rest of the distribution, except for points that are determined to be outliners (displayed as dots) using a method that is a function of inter-quartile range.

Structural Similarity Profile

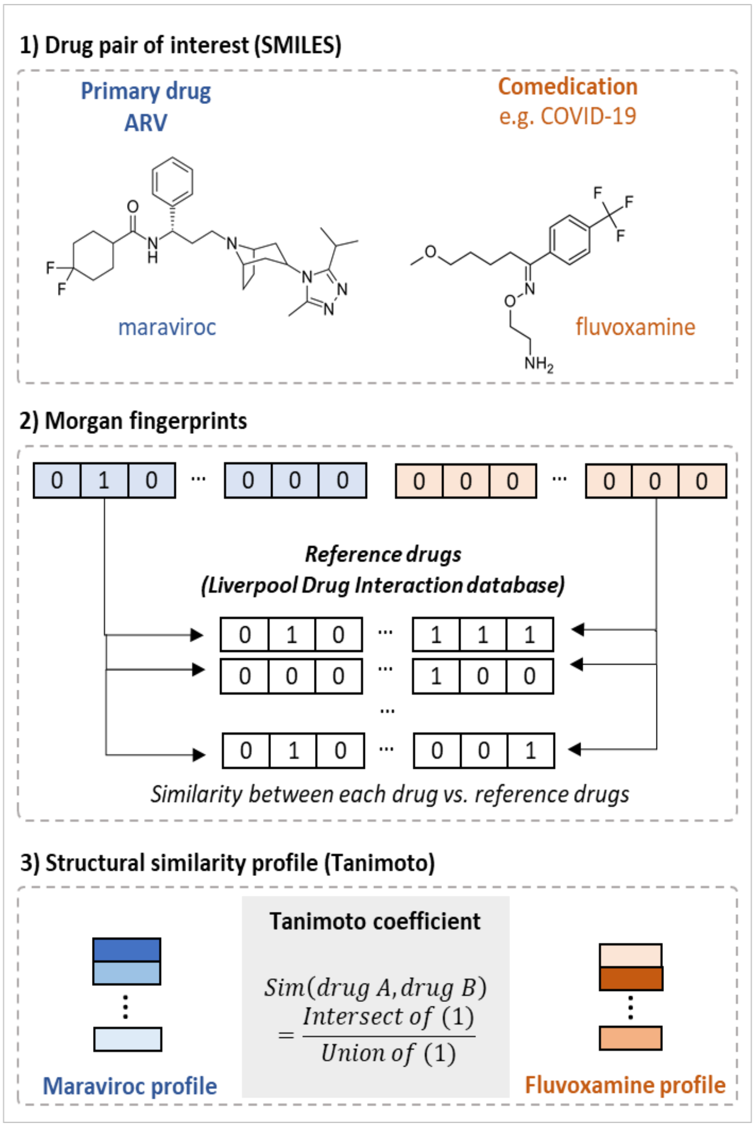

Supplementary Figure 2. Overview of structural similarity profile framework. 1) Compound structure in the form of SMILES for a primary ARV and a comedication are extracted from PubChem. **2)** SMILES are converted into Morgan binary fingerprints, and **3)** their similarities are measured using Tanimoto coefficients. The similarity profiles between two drugs are fed into DeepARV as input features.

Test Set

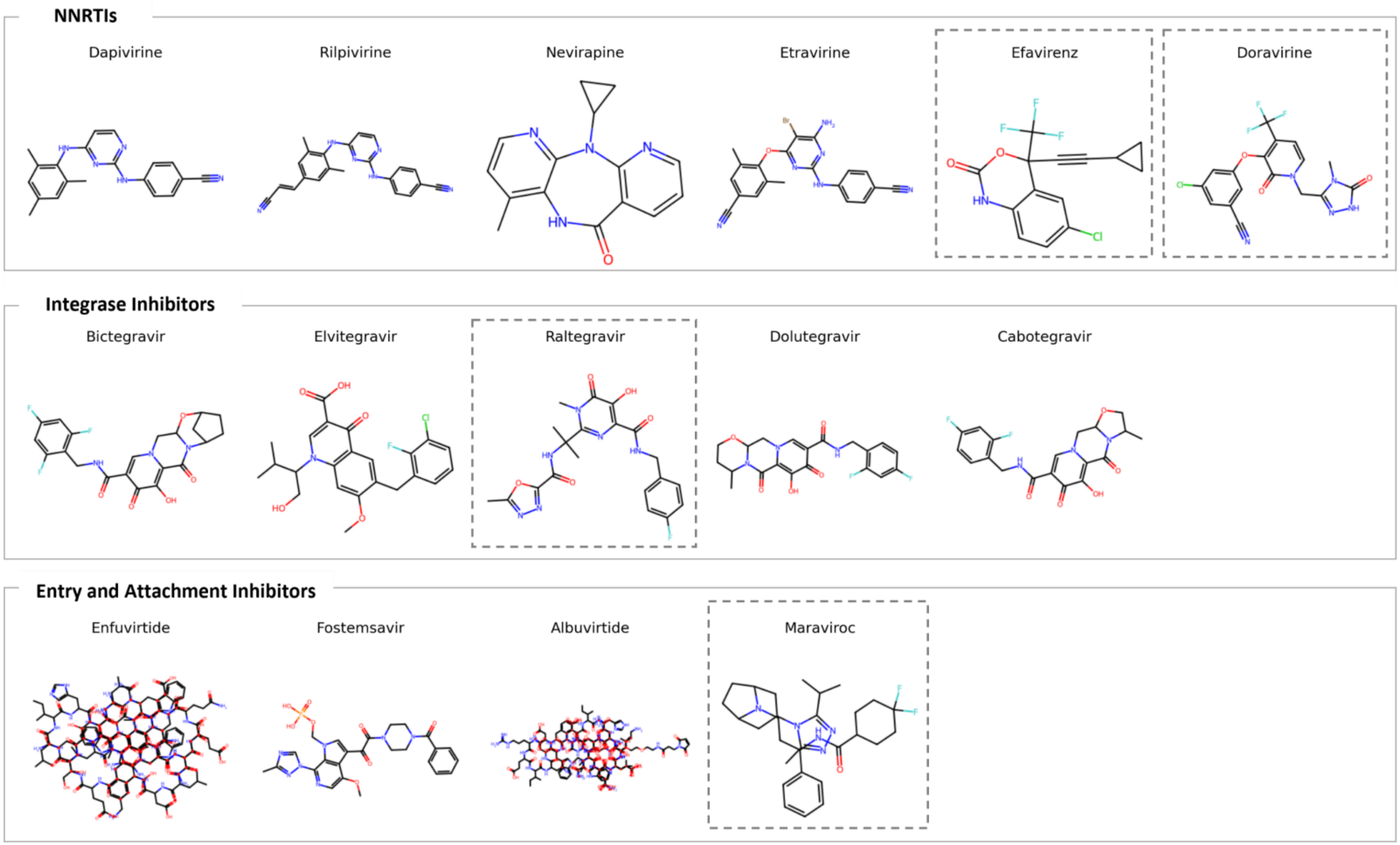
NNRTIs
Dapivirine
Rilpivirine
Nevirapine
Etravirine
Efavirenz
Doravirine
Integrase Inhibitors
Bictegravir
Elvitegravir
Raltegravir
Dolutegravir
Cabotegravir
Entry and Attachment Inhibitors
Enfuvirtide
Fostemsavir
Albuvirtide
Maraviroc

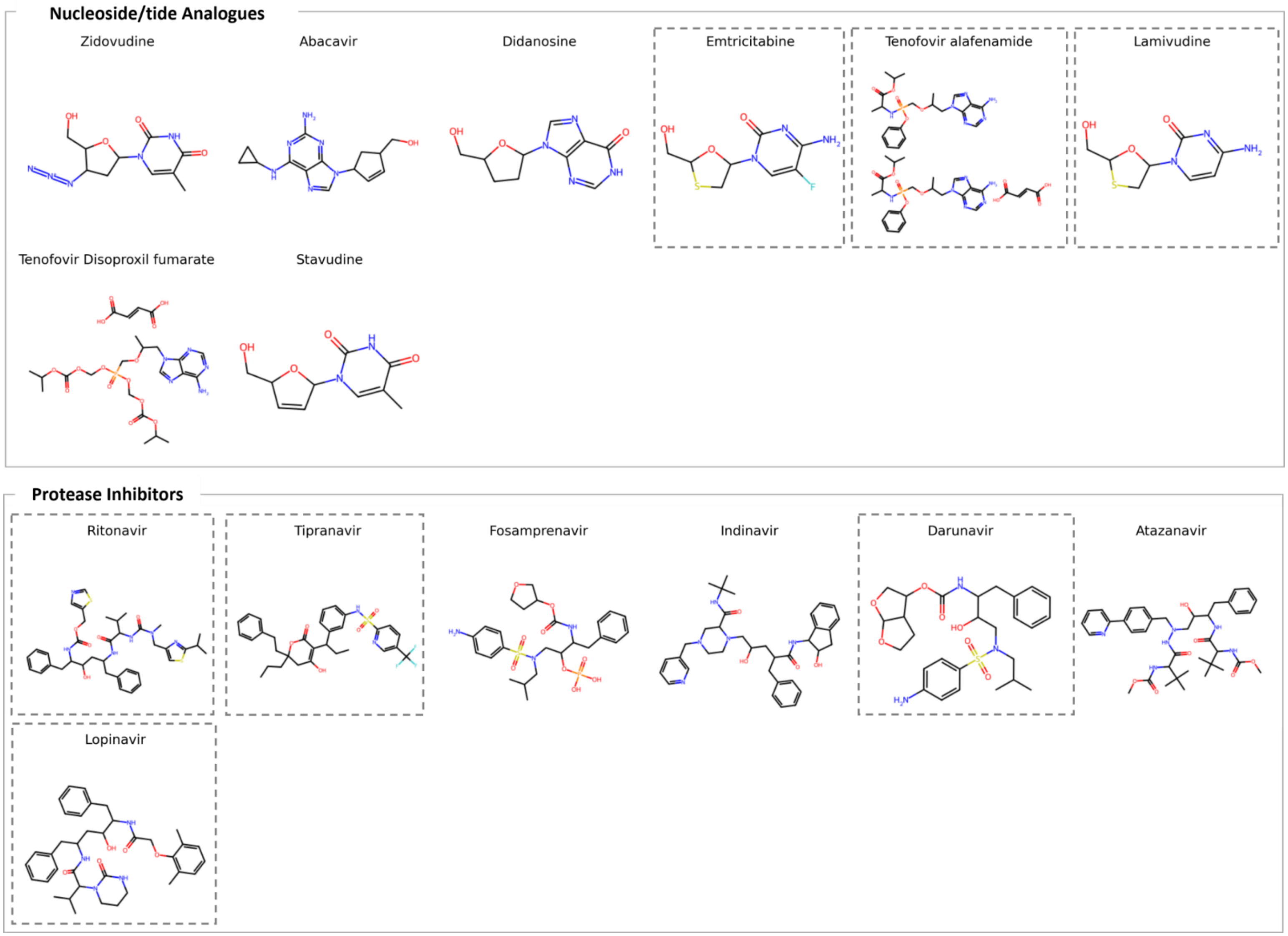

Supplementary Figure 3. Overview of ARVs distinct drug classes and structures. Dash-lined boxes indicated the drugs selected for the independent test set.

Optimisation of the neural network

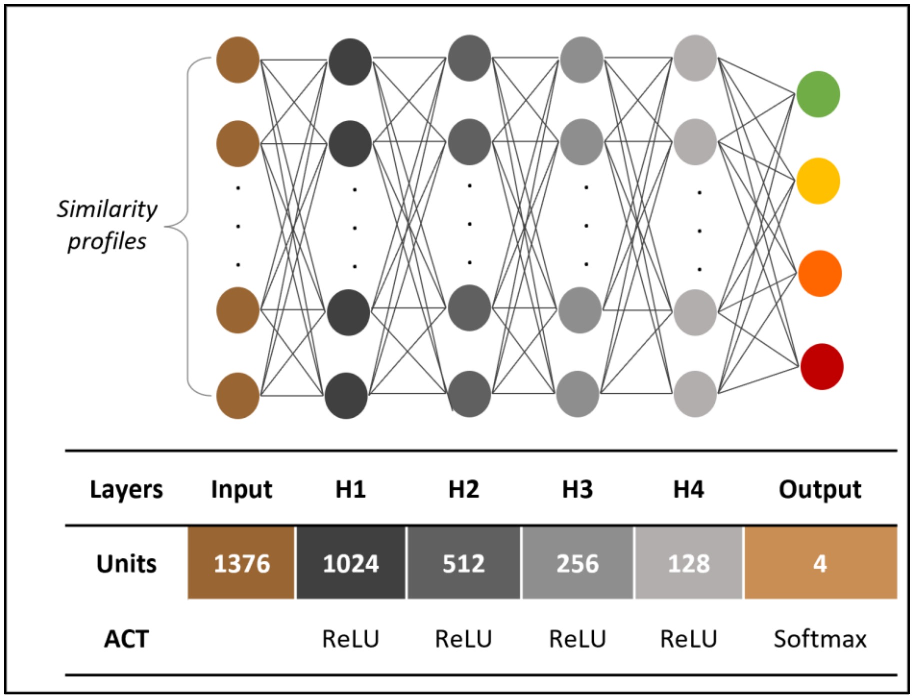

Supplementary Figure 4. The optimal architecture of DeepARV-Sim. It is composed of an input layer with 1376 nodes length, four hidden layers of 1024, 512, 256 and 128 neurons respectively, each was followed by a dropout layer of rate of 0.2, and four output nodes of Green, Yellow, Amber and Red DDI types. The activation functions used between input and hidden layers were ReLU and between final hidden layer and the output was Softmax.

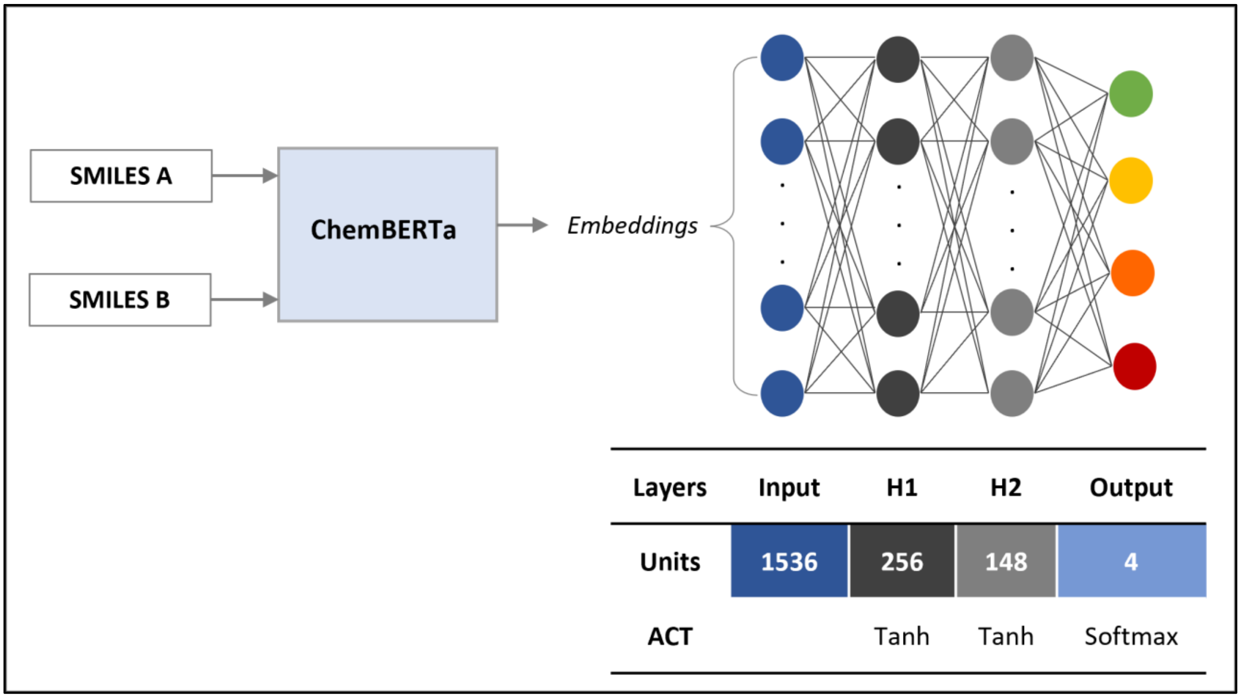

Supplementary Figure 5. The optimal architecture of DeepARV-ChemBERTa. Given drug SMILES, ChemBERTa featuriser output the embeddings which were concatenated for a drug pair and fed into the input layer with 1536 nodes length. The optimal architecture of subsequent neural network is composed of two hidden layers of 256 and 128 neurons respectively, each was followed by a dropout layer of rate of 0.2, and four output nodes of Green, Yellow, Amber and Red DDI types. The activation functions used between input and hidden layers were Tanh and between final hidden layer and the output was Softmax.
